# How Encephalopathy Impacts Language Ability: A Scoping Review of the Linguistic Abilities of Adults with Developmental and Epileptic Encephalopathy

**DOI:** 10.3390/medicina60101635

**Published:** 2024-10-06

**Authors:** Ioanna Papatheodorou, Stavroula Stavrakaki, Vasiliki Koukoulioti, Martha Spilioti, Vasileios Kimiskidis

**Affiliations:** 1 School of Medicine, Aristotle University of Thessaloniki, 546 36 Thessaloniki, Greece; idpapathe@auth.gr (I.P.); marthags@auth.gr (M.S.); 2 Department of Italian Language and Literature, School of Philosophy, Aristotle University of Thessaloniki, 546 36 Thessaloniki, Greece; svoula@itl.auth.gr; 3 Department of German Language and Literature, School of Philosophy, Aristotle University of Thessaloniki, 546 36 Thessaloniki, Greece; vasilikikou@del.auth.gr

**Keywords:** Landau–Kleffner syndrome, Dravet syndrome, continuous spikes and waves during slow sleep, language impairment, cognitive impairment

## Abstract

*Background and Objectives*: Developmental and epileptic encephalopathy refers to a group of conditions where patients experience abnormal development due to various causes as well as frequent epileptiform discharges that ultimately contribute, in an independent and additive fashion, to cognitive and linguistic impairments. The language and cognition outcome of these patients in adulthood has been understudied. This paper aims to present a scoping review of linguistic abilities in adults with developmental and epileptic encephalopathy to determine the extent to which language outcomes in adulthood and their relation to cognitive outcomes have been studied. *Design*: Two online databases were searched and the methodological framework by Arksey & O’Malley (2005) was adopted. *Results*: Out of the 27 selected studies, only 13 exclusively examined adults, 15 were group studies, 5 were case studies and 7 were case series. A total of 9 out of the 15 group studies provided individual results for adults. Twenty-two studies included a follow-up examination. Twenty-three studies addressed the relationship between language and cognition. The selected studies indicate the presence of language impairments, which are nevertheless differentially manifested in the syndromes under investigation, whereas individual variability is also reported. Aspects of cognition seem to correlate with linguistic abilities. *Conclusions*: In sum, despite variability in linguistic abilities, language deficits constitute a significant aspect of the clinical profile of many adults with developmental and epileptic encephalopathy, a finding that should be taken into account for the treatment protocols of these individuals.

## 1. Introduction

The term epileptic encephalopathy (EE) was introduced in the 1950s by Landau and Kleffner, and was further used by Gastaut et al. in the 1960s in order to describe certain cases in patients where epilepsy would arise (predominantly) in childhood [1]. The term has since evolved and been used to describe different conditions over the years. The first definition proposed by the International League Against Epilepsy (ILAE) described EE as a condition in which epileptic activity is “believed to contribute to the progressive disturbance in cerebral function” [2] (p. 979). In this same vein, Berg et al. [3] has described epileptic encephalopathy as a cognitive and behavioral impairment, which is caused by epileptic activity and exceeds what would be expected based on the cause of the epilepsy alone (e.g., structural–metabolic causes). In the classification proposed by the ILAE in 2017, a distinction between epileptic encephalopathy, developmental and epileptic encephalopathy and developmental encephalopathy was proposed [4]. More specifically, it was suggested that epileptic encephalopathy (EE) should be used to refer to cases of encephalopathy due to epilepsy in all ages and not be restricted to certain syndromes of encephalopathy associated with seizures in infancy and childhood [4]. The defining characteristic of EEs are the significant cognitive and behavioral impairments that are derived primarily as the result of frequent epileptiform discharges.

Developmental and epileptic encephalopathy (hereafter DEE), on the other hand, has been used to describe conditions that are characterized by: (a) developmental delays due to various factors, including genetic factors, that manifest before seizure onset; and (b) by significant epileptic activity that acts as an independent and additive causative factor for the cognitive and behavioral impairments of these patients. In some cases, the seizures appear so early on in life that the two factors cannot be disentangled from each other. Finally, the term developmental encephalopathy is reserved for cases in which “there is just developmental impairment without frequent epileptic activity associated with regression or further slowing of development” [4] (p. 519). It should be mentioned that as far as the present study is concerned, we will use the term epileptic encephalopathy and developmental epileptic encephalopathy interchangeably. The reason for this is that the term DEE has been established only in the last 7 years and therefore was not incorporated in older literature.

It is crucial to highlight that the exact mechanisms driving epileptic encephalopathy remain unclear, but they probably involve dysfunction across widespread neuronal networks. While there is some overlap among these syndromes, pinpointing a specific electroclinical syndrome is of utmost importance, as it directs treatment decisions and provides insights into the prognosis [5].

Six syndromes which occur during infancy and childhood traditionally meet the criteria for epileptic encephalopathies. These are the Dravet syndrome; Doose syndrome (currently known as EMAtS, Epilepsy with myoclonic atonic seizures); electrical status epilepticus of slow sleep (ESES) or continuous spike waves of slow sleep (CSWS); Landau–Kleffner syndrome; Lennox–Gastaut syndrome; Ohtahara syndrome (nowadays falling within the spectrum of early infantile DEE); and West syndrome (currently described in the context of infantile epileptic spasms syndrome (IESS [6,7,8]). Some researchers also include severe epilepsy with multiple spike foci in the group of epileptic encephalopathies (see Parisi et al. [6] and references therein). In this review, we included only those syndromes for which studies on language were detected through our search, specifically the continuous spike waves of slow sleep syndrome, the Landau–Kleffner syndrome and the Dravet syndrome.

### 1.1. Continuous Spike–Waves during Slow Sleep Syndrome (CSWS)

Continuous spike-waves during slow sleep syndrome (CSWS) is a childhood epilepsy that was first reported by Patry, Lyagoubi and Tassinari [9], who described a peculiar EEG pattern with paroxysmal abnormalities in six children that occurred almost continuously during sleep. The EEG pattern was characterized by “subclinical” spike and wave complexes lasting from months to years, with these spike–waves occupying at least 85% of NREM sleep [9]. The term “ESES” (electrical status epilepticus during sleep) was introduced and associated with epilepsy, mental deficits and delayed speech [9]. The term “continuous spikes and waves during slow sleep” (CSWS) was introduced in 1989 when the ILAE included CSWS in a group of epileptic syndromes that cannot be determined as generalized or focal [10]. Atypical absences, myoclonic and atonic attacks, as well as generalized tonic–clonic seizures may be present, although not all patients with CSWS have seizures [11,12]. ESES and CSWS are often used interchangeably, although ESES points towards the EEG patterns, whereas CSWS indicates the electroclinical aspects of the syndrome [13]. CSWS only develops in the first 10 years of life [13]. The current classification approach to DEEs introduced the umbrella terms DEE–SWAS and EE–SWAS in order to describe the cluster of syndromes characterized by cognitive, linguistic and behavioral deficits and motor regression as well as prominent spike and wave activation during sleep [14]. This term has encompassed and replaced the formerly used term “epileptic encephalopathy with continuous spike-and-wave in sleep”. It should be also noted that EE–SWAS contains as a distinct subtype the Landau–Kleffner syndrome, characterized by marked language regression due to an acquired auditory agnosia (vide infra) [14]. For the needs of the current study and in order to include older relevant literature, CSWS was employed.

CSWS is associated with cognitive deterioration and behavioral disorders. Many authors have associated CSWS with language deficits, and the cognitive and linguistic impairment can either be present as global or selective [15,16,17,18,19,20]. Motor impairment, including ataxia and dyspraxia has been observed in some patients [18,21] as well as a decline in intelligence quotient (IQ) or developmental quotient (DQ), attention deficit, hyperactivity, aggressiveness, psychosis, anxiety and autistic traits [13,15,18,20].

Family history of epilepsy is generally uncommon, being mentioned in about 10–15% of the cases [13,22,23]. In approximately two-thirds of cases, neuropsychological and psychomotor development is typical before CSWS onset. In the remaining cases, language-related delays are more prominent [22]. Structural defects have been found, including pre- or perinatal vascular lesions and cortical malformations [24,25]. Accordingly, neuropsychological deficits before CSWS onset may include psychomotor and mental retardation, fixed encephalopathy and congenital hemiparesis [13,23].

### 1.2. Landau–Kleffner Syndrome

In the past, there was much controversy surrounding the clinical differential diagnosis of CSWS and LKS. In particular, some authors had noticed that CSWS and LKS passed into one another or that reported cases evolved from LKS to CSWS, suggesting that the two syndromes are likely different manifestations of a shared, unidentified cerebral dysfunction [18,26]. While interesting, this discussion was considered out of the scope of the present review to the extent that it was not relevant to the language performance of individuals with shared dysfunctions. Nowadays, LKS is considered a particular subtype of EE–SWAS.

Landau–Kleffner syndrome was first described by Landau and Kleffner in 1957, who reported the case of six children with various types of seizures and acquired aphasia [27]. Age of onset is typically between 3 and 8 years old [13,28]. It occurs in children with previously normal neuropsychological and cognitive development, who had already developed language abilities appropriate for their age. It is not associated with brain lesions, as the patients present with normal structural imaging, and should be differentiated from retarded language function, often linked to developmental delay [13,23,28,29,30]. The cause of their condition is unclear and inherited genetic factors are not considered significant contributors [30].

The primary clinical manifestation of LKS is language regression and progressive aphasia over weeks up to months, with auditory agnosia progressing as well during this period. Due to auditory agnosia, children are unable to understand speech and meaningful sounds, and in some cases, they present with a complete loss of understanding and verbal communication, which makes them appear as deaf-mute, even though the audiograms are largely normal [13,23,28,29,30,31]. Some authors have linked the inability to distinguish sounds with a specific deficit in phonological processing and decoding [32,33,34], whereas others have posited that it reflects a more general auditory processing deficit [34,35,36,37]. Impaired short-term auditory memory is also a consistent finding among studies, even when the patients show relatively good outcomes [32,35,38].

Most patients have presented with auditory agnosia, which is nevertheless manifested prior to expressive linguistic limitations and epilepsy [30]. Not all patients present with seizures, which are typically infrequent, easily treated, self-limited and decline in frequency with age. Different types of seizures are possible, including tonic–clonic, atypical absences and, more rarely, myoclonic astatic seizures [13,28,29,30]. EEG is typically activated with sleep, and an EEG pattern of ESES is commonly observed in many LKS cases. Paroxysmal spike and slow waves are present, which are near continuous during slow wave sleep. The spike foci are typically found in temporal areas and the discharges are either unilateral or bilateral [13,29,30]. Other clinical symptoms may include behavioral disturbances and psychiatric symptoms such as irritability, hyperkinesia, attention deficit and autistic traits [28,29,39].

### 1.3. Dravet Syndrome

Dravet syndrome (DS) appears in the first year of life in previously typically developing infants. It is characterized by febrile seizures, and later afebrile seizures, primarily focal unilateral clonic seizures of prolonged duration, as well as generalized tonic–clonic seizures [40,41]. Status epilepticus is common and other seizure types include myoclonic or atypical absence seizures. The seizures are pharmacoresistant, even though epilepsy severity declines as patients move from childhood to adolescence and adulthood. During the second year, developmental and cognitive delay becomes apparent [40,41]. Two forms of DS are currently identified, typical DS and borderline DS, the latter being characterized by a similar clinical profile and outcome but by the absence of myoclonic seizures [40,41,42,43]. A SCN1A gene mutation causing deterioration of function is found in at least 80% of the patients [7,44]. Family history of epilepsy or febrile seizures and SCN1A mutations have been reported in approximately 25–71% of patient cases [40,41]. Various authors have reported fatalities in DS patients, frequently at a young age, and the mortality rate is high, at approximately 16% [40,41,45,46]. There is also an increased risk of sudden unexplained death in epileptic patients (SUDEP) [7,40,41].

Regarding cognitive and developmental deterioration, children normally start acquiring walking and language skills at the expected age, but a characteristic crouch gait develops and persists for an extended period. Language acquisition is delayed, often resulting in difficulties forming basic sentences [40]. Fine motor skills are underdeveloped, and behavioral issues, which contribute significantly to learning disabilities, are present [40,42,44,47,48]. Almost invariably severe mental impairment and poor cognitive outcome in adulthood have been reported by some studies [42,48], but the majority of the studies support cognitive deficits “relatively homogeneous in quality but of different degrees” [40] (p. 6), depending on various factors [44,45,46,48].

### 1.4. Summary and the Motivation for the Present Scoping Review

Developmental and epileptic encephalopathies affect the typical development of language among other cognitive functions. As childhood constitutes a critical period for language acquisition, abnormal electrical activity during this developmental window could disrupt the typical course of acquiring linguistic abilities [29] and lead to long-lasting language deficits. Despite extensive research in the field of DEEs and EEs, the language profiles of patients in adulthood and long-term linguistic outcomes have not received sufficient attention in the literature. Furthermore, there are scarcely any studies that adopt a comparative approach, i.e., studies that investigate similarities and differences in the linguistic profile among patients with DEE/EEs, which is necessary in order to investigate the overall long-term effect of EE manifested in infancy and childhood. Related to this, it is not clear how selective the impairment in each syndrome is; in other words, which modality and/or which linguistic level is affected in the different syndromes. Last but not least, the patterns of deterioration and/or improvement in the course of the disease across syndromes have not been systematically investigated. A nuanced understanding of language-related challenges is crucial for both clinicians and researchers and can have implications for diagnostic considerations, intervention strategies and future research directions.

For these reasons, a scoping review was conducted in order to explore the available literature regarding the language profiles of adults with (developmental) and epileptic encephalopathy. This review aims not only to systematically map the existing research on language deficits in adults with EE, but also identify deficient areas of understanding of the linguistic abilities of adults with EE and, consequently, gaps in our knowledge of individuals with EE. In particular, four research questions have been addressed:

1. Are there language deficits in adulthood across LKS, CSWS and the Dravet syndrome?
2. If yes, in which modalities (oral production/oral comprehension/written production/written comprehension) and at which linguistic levels (pragmatics, phonology, morphosyntax, etc.) do they manifest?
3. Do these syndromes share the same manifestations of linguistic deficits or do they differ?
4. Are there any cognitive correlates of linguistic impairments in individuals with DEE/EE?

## 2. Methodology

The methodological framework described by Arksey & O’Malley [49] for conducting scoping reviews has been adopted and the Preferred Reporting Items for Systematic Reviews and Meta-Analyses (PRISMA) guidelines have been followed. The PRISMA checklist has been completed in order to ensure compliance with the established guidelines (see Table S1 in the Supplementary Material). A pilot search was carried out to identify pertinent keywords for the subsequent comprehensive search. It was decided to use different search terms for each syndrome, because they are characterized by different language symptoms, as shown also in the pilot search. Articles which fulfilled the following criteria were included. The eligible articles should: (1) refer to (at least) one of the three epileptic encephalopathy syndromes (Dravet, CSWS or LKS); (2) investigate language impairment in adulthood; (3) report original research; (4) be written in English; (5) be published in peer-reviewed scientific journals; and (6) be published after 2000. To identify relevant articles, PubMed and Scopus were searched from December 2023 to January 2024. Last access was performed in September 2024 for validation. The studies included all language domains (pragmatics, phonology, syntax and grammar) and all language modalities (oral production, oral comprehension, writing and reading) as well as studies relating language to metalinguistic and language-related cognitive abilities. Language evaluations could be based either on standardized linguistic tests or clinical evaluations and observations, whereas cognitive evaluations were required to be conducted through standardized neuropsychological tests.

The final search terms for CSWS in the Scopus database were: TITLE-ABS-KEY (“CSWS” OR “Continuous Spikes and Waves” OR “Continuous Spike-Waves”) AND ALL (“language” OR “long term follow-up” OR “language impairment” OR “cognitive impairment” OR “communication abilities”) AND ALL (adult*) AND PUBYEAR > 1999 AND PUBYEAR < 2024 AND (LIMIT-TO (DOCTYPE, “ar”)) AND (LIMIT-TO (LANGUAGE, “English”)). In the PubMed database, the search query was: (((“Continuous Spike and Waves” [Text Word]) OR (“CSWS” [Text Word])) OR (Continuous Spike-Waves [Text Word])) AND (“language” OR “language impairment” OR “cognitive impairment” OR “long term follow-up” OR “communication abilities”) AND “adult*”. Similarly, for the search about LKS syndrome in Scopus, we used the query: TITLE-ABS-KEY (“Landau–Kleffner syndrome”) AND ALL (“language” OR “long term follow-up” OR “language impairment” OR “cognitive impairment” OR “speech disorders” OR “verbal auditory agnosia”) AND ALL (adult*) AND PUBYEAR > 1999 AND PUBYEAR < 2024 AND (LIMIT-TO (DOCTYPE, “ar”)) AND (LIMIT-TO (LANGUAGE, “English”)). In PubMed, the query was: (“Landau–Kleffner syndrome” [Text Word]) AND (“language” OR “language impairment” OR “cognitive impairment” OR “long term follow-up” OR “communication abilities” OR “speech disorders” OR “verbal auditory agnosia”) AND “adult*”. Finally, for Dravet syndrome, the search terms in Scopus were: TITLE-ABS-KEY (“Dravet syndrome” ) AND ALL (“language” OR “long term follow-up” OR “language impairment” OR “communication abilities” OR “cognitive impairment” OR “speech disorders” OR “oral motor skills”) AND ALL (adult*) AND PUBYEAR > 1999 AND PUBYEAR < 2024 AND (LIMIT-TO (DOCTYPE, “ar”)) AND (LIMIT-TO (LANGUAGE, “English”)) and in PubMed (“Dravet syndrome” [Text Word]) AND (“language” OR “language impairment” OR “cognitive impairment” OR “long term follow-up” OR “communication abilities” OR “speech disorders” OR “oral motor skills”) AND “adult*”.

The final search results were exported to Zotero [50] (https://www.zotero.org/) and subsequently to web-tool rayyan [51] (https://www.rayyan.ai/). Duplicates emerging from the search in two databases were identified and removed in Zotero for each syndrome separately. The rayyan tool was used in order to assess agreement among the authors concerning the reports assessed for eligibility. The three first authors (IP, SS and VKO) voted for inclusion or exclusion independently of each other. In the few cases of disagreement, they were resolved after discussion.

In the first screening on the basis of the title, we identified for each syndrome: (1) articles that did not have epilepsy as a topic (we found false hits for CSWS); (2) articles that were totally unrelated to language or cognition in epilepsy (e.g., articles on genetics, treatment, motor skills or behavioral profiles); and (3) articles that were reviews. After removing these three categories, in a second screening (based on abstract reading), we identified and removed the studies that: (1) did not include any adult participants; (2) did not have any focus on language; and (3) were general overview/positional papers. Other reasons for excluding in this step were, for example, those containing no clear diagnosis, or reports of mixed results for more than one syndrome. The remaining eligible papers were reviewed. Finally, after detecting the eligible articles for each syndrome, we performed a final duplicate detection in the set of eligible articles, as some articles were in the results list of more than one syndrome searches.

For the review process, a data charting form was developed jointly by the three first authors (IP, VKO and SS). The data extracted from each study were the following: (1) number of participants; (2) age of participants; (3) language of the participants; (4) data collection method; (5) materials used; (6) language modality and language abilities assessed; and (7) the findings concerning language performance in adults with epileptic and developmental encephalopathy. We grouped the studies by syndrome and summarized for each one the information charted as described.

## 3. Results

### 3.1. Study and Participant Characteristics

**Figure 1:**
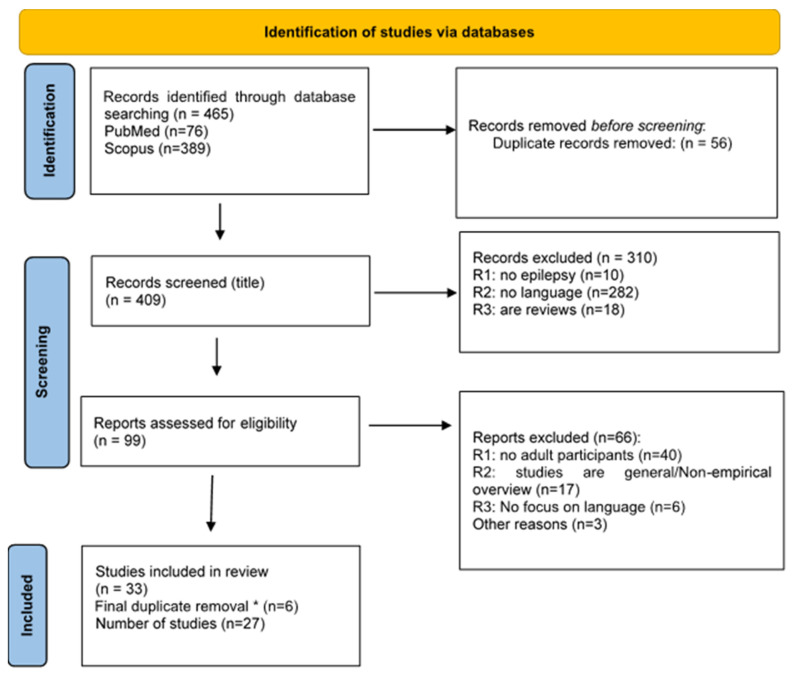
PRISMA flow diagram. * We performed a duplicate removal after merging all the eligible papers resulting from the searches for each syndrome separately.

As shown in the PRISMA flowchart (Figure 1), 27 studies met the inclusion criteria. A total of 11 articles studied LKS, 8 studied DS, 7 studied CSWS and 1 addressed two syndromes (CSWS and LKS).

Almost half of the studies (13/27) included only adults, whereas the rest (*n* = 14) tested both adults and children/teenagers. Overall, 519 participants (children and adults) were included in the selected studies, whereby only 186 participants were adults. However, this number is probably higher, as four studies with large cohorts did not report the number of adult participants. Moreover, one adult in the study by Veggiotti [20] is also reported in Veggiotti [19], though not explicitly declared, and some studies consider 16 years old the age limit for adulthood. The age range of the adult participants was relatively large (16–50 years). Concerning the languages studied, the most studied one is English (*n* = 8), followed by Italian (*n* = 6). Two studies had French-speaking participants and there was one study for Norwegian and one for Swedish. Interestingly, for a third of the studies (*n* = 9) no language is mentioned. Most studies concern both production and comprehension (*n* = 16), six studies concern only production, two studies concern only comprehension and three studies refer to language in general, as part of a neuropsychological examination without further specification. Concerning the study design, 15 studies concerned groups, 5 were case studies and 7 were case series. Out of the 15 group studies, only 9 provide individual results. Furthermore, most studies (*n* = 23) addressed the relation of language with cognition. Finally, the majority of the studies (*n* = 22) included a follow-up examination. Concerning the terminology, it is interesting that only two-fifths of the studies published after 2017 used the term developmental and epileptic encephalopathy. A summary of study characteristics is presented in Table 1. Visualization of the study characteristics including the number of languages, number of group studies, case series and case studies as well as the number of studies for different syndromes, the number of studies for different modalities (production, comprehension, etc.) as well as the number of studies testing each modality in each syndrome are presented in Figures S1–S7 in the Supplementary Material. All reviewed papers and their results are presented in Table S2 in the Supplementary Material. 

### 3.2. Synthesis of Results

The papers included in our study (see Table 1) are analyzed as follows: First, with respect to the language impairments shown in adults with CSWS, LKS and DS; second, with respect to the cognitive correlates of the manifested linguistic deficits. The most significant points of this analysis are summarized in Table 2.

#### 3.2.1. Language Deficits in Adulthood in CSWS, LKS and Dravet Syndrome

##### CSWS

Language deficits in CSWS manifest predominantly in the domain of semantics and pragmatics. Some studies report grammatically (phonologically and syntactically) correct language often devoid of content [19,20], semantic paraphasias [20], poor lexical fluency [16,33] or poor language semantic fluency [52] and logorrhea with pragmatic disorders [33]. Performance in naming, which also touches on semantics, has been reported to be preserved, nevertheless [15,16,17,33]. Interestingly, in the case study by Hommet et al. [16], the same patient had poor lexical fluency but preserved naming. While patients are reported to produce phonologically and syntactically correct language, as mentioned above, there is evidence for syntactic deficit in spontaneous speech [16]. Debiais et al. [15] reports two adults, one with impaired and one with spared syntactic production.

Concerning input modality, only one study examined lexical judgement and found severe impairment for one patient and normal performance for another [15]. Other studies discuss general comprehension abilities and report deficits for at least for some [33], residual deficits for others (with no further specification of the nature of the deficit) [17], while others provide evidence for deficits in the comprehension of more complex sentences but not of simple ones [16]. The patients of Debiais et al. [15] showed normal performance in language comprehension. Nevertheless, the patient who was impaired in expressive grammar was also impaired in grammaticality judgement, whereas the other patient had intact performance in both tasks.

Regarding written language, CSWS patients have been reported to manifest dyslexia and dysgraphia [19,33] and in general present with poor performance in reading and writing [16]. Seegmüller et al. [17] report large variability among their patients (with two achieving basic reading and writing, four achieving good abilities, one manifesting dyslexia and one having no abilities at all). Similarly, one patient in the Debiais et al. study [15] was impaired both in spelling and reading, whereas these abilities were spared for the other.

All in all, except for naming, which has been consistently found to be spared in CSWS, there is a large individual variability. Furthermore, no modality specific or linguistic level specific deficits have been observed in oral language. Despite the fact that there is a large variability in written language, too, a symmetry has been observed, as both reading and writing are either spared or impaired in each patient. Finally, Pera et al. [53] reported language improvement coinciding with the regression of EEG abnormalities in their study. Nevertheless, it was not mentioned whether this finding concerns adults.

##### Landau–Kleffner Syndrome

LKS is characterized by the sudden or gradual development of auditory agnosia, which is the inability to understand spoken language and semantically interpret and differentiate distinct sounds [35,54,55,56]. However, the language abilities of individuals with LKS vary in adulthood. In fact, many patients achieve normal language abilities in adulthood [35,37,54,55], while others manifest persistent mild to moderate language disorder [35,37,54,55,57], and still others present with severe language disorder [35,37,55,56], being unable to produce oral language altogether [34]. The outcome seems to be independent of the severity of the verbal auditory agnosia in childhood, as in the study of Caraballo et al. [54] all but one had had severe agnosia in childhood, but mild/moderate language disorder as adults. The group studies indicate that most patients had normal language abilities or were mildly affected in adulthood (6 out of 13 in Caraballo et al. [54], 3 out of 5 in Cockerel et al. [35], 3 out of 4 in Duran et al. [55] and 4 out of 8 in Rejnö-Habte Selassie et al. [37]), whereas only a few were severely impaired (2 out of 13 in Caraballo et al. [54], 0 out of 5 in Cockerel et al. [35], 1 out of 4 in Duran et al. [55], and 4 out of 8 in Rejnö-Habte Selassie et al. [37]).

Concerning speech production, mildly to severely impaired oral motor abilities, stuttering and articulatory deficits/apraxia have been reported [34,37,57]. Word finding difficulties have also been reported for some patients [54,57,58] in confrontation naming but also in phonologically driven retrieval [37] or verbal fluency tasks [33]. Errors consist of semantic and phonemic paraphasias [34,54,57]. Large variability has been observed also in sentence production, as studies indicate normal or mild/moderate impairment [37] but also severe impairment of expressive grammatic abilities [37,58].

Concerning language receptive abilities, there is evidence for impaired prosody processing [38] and impaired phonological discrimination [34] in some patients, while others are not affected [38]. Variable outcomes are observed also in receptive vocabulary and grammar, with some patients manifesting severe deficits [34,37,58], often resulting in an inability to perform the tests altogether [33] while others have mild or moderate deficits [37,57].

Variability in overall communication is observed among the literature, with some authors reporting moderate to severe communication problems, leading even to mutism [54,55], while others report good communication [35,38]. Despite persistent language disorder in some patients, some authors refer to functionally independent LKS patients who still relied on lip-reading, eye contact or sign language in order to understand oral speech due to comprehension issues [34,56,57].

Concerning written language, the same variability has been observed with some patients achieving a normal level of performance [36] and others facing severe difficulties [34,37,57], or an inability to complete tasks like text reading and dictation [33]. Slow oral reading and misreading of unfamiliar words have been reported as problems [57], whereas one patient was found to face difficulties with phonetic reading compared to visual word recognition [34].

##### Dravet Syndrome

There is variability when it comes to oral production in DS, with some patients being able to speak a few words indicating poor but communicative language, while the majority exhibit structurally impaired language, an inability to communicate and produce intelligible speech and even no speech at all [45,46,59]. Among the cohort of 31 patients by Akiyama et al. [45], only 5 of them were able to have simple conversations and partially read. Among the 24 patients assessed by Genton et al. [46], only 7 of them had communicative speech. Turner et al. [59] distinguished their 20 patients into a minimally verbal and a nonverbal group, and all but 1 adult patient had scores below the mean; apart from 1, the other 3 verbal adults had moderate to severe conversational speech intelligibility. Darra et al. [42] examined 50 adults with DS, classifying them in ‘complete’ and ‘incomplete’ phenotypes, the latter referring to DS without atypical absences and myoclonic seizures. Cases of normal language and simple conversations were reported among the patients, but the majority of the participants were able to produce only short sentences or single words, while some of them had no speech at all [42].

Impaired articulation of consonants and vowels, unusual nasal tone, breathy, strained and low-pitched voice, as well as errors in prosody, characterized the speech production of DS patients [59]. A significant impairment concerning expressive abilities in DS patients was also found in oral motor skills. Turner et al. [59] examined dysarthria among other motor speech deficits in DS. They found that all patients had impaired oral motor control and there were limitations, irregularities or ineffective coordination in lip and tongue movements. Challenges in motor programming and planning significantly impacted the execution of both speech and non-speech oral motor tasks. The authors noted that altered inhibition in DS patients may also impact speech and language [59]. DS patients have also been reported to display parkinsonian features, including dysarthria, and there is an observed trend of increasing severity of these symptoms with the onset of age [60]. The first case experienced crouch gait, dysarthria and intellectual disability from age 6. Her language was minimally expressive and communicative and she exhibited bradykinesia, ataxia and tremor by the age of 18. The second case experienced tremor, intellectual disability and developed dementia in her 30s, with pharmacological treatment improving her communication but not affecting seizure frequency. The authors decided that the origin of parkinsonism in DS remained uncertain, with unclear links to severe SCN1A gene mutations or persistent seizures treated with various high-dose AEDs [60].

Our research did not yield studies addressing receptive language and comprehension deficits in DS in adults. Brown et al. [61] assessed adult patients with DS as a subset of a larger cohort with NEPSY which tested for comprehension of instruction and vocabulary. They also used the Delis–Kaplan Executive Function System (D-KEFS)—Verbal Fluency to measure phonetic and semantic fluency, as well as category switching, but no specific information on the relation between language and cognition or individual results for adults are provided.

Summary: Input and Output Language Abilities in Adulthood Across Syndromes

While the significant variation attested within and between syndromes makes a summary a significantly difficult enterprise, an attempt has been made to summarize the main points in terms of the input and output modality (receptive and expressive speech–language abilities) across syndromes.

Summary I: Receptive Abilities

All syndromes show difficulties in receptive abilities. CSWS patients are found to have limitations in written language comprehension and oral language comprehension, particularly sentence comprehension. They also show impaired lexical and/or grammatical judgment abilities. LKS patients present with difficulties in oral and written language reception as they show impairments in spoken language comprehension, distinct perception of sounds and phonological discrimination and awareness. Finally, our research yielded only one study addressing receptive language and comprehension deficits in DS in adults, which suggests an impairment. In our view, while deficient performance has been reported, accurate conclusions concerning identical performance patterns cannot be made.

Summary II: Expressive Abilities

All syndromes have deficient expressive abilities. CSWS patients show striking semantic and pragmatic difficulties as well as deficient lexical fluency. LKS patients are characterized by simplified and short utterances, word retrieval difficulties, paraphasias and cluster and syllable reduction. DS patients show severe production deficits as they either appear able to produce only short sentences/single words only or articulate no speech at all. Apparently, the deficient domains across syndromes are not identical.

#### 3.2.2. Cognitive Correlates of Language Abilities

##### Continuous Spike–Waves during Slow Sleep Syndrome

A dysexecutive syndrome has been described during the active phase of CSWS. The pragmatic impairment reported in these patients seems to be brought into interplay with executive functions by various authors [15,16,20,33]. However, Praline et al. [33] pointed out that there was not clear evidence in their study supporting a dysexecutive syndrome, as the neuropsychological disorders observed, such as pathologic verbal fluency, uninhibition and inattention, were only seen in two of their patients. Notably, all of their patients exhibited a pragmatic impairment, which could be interpreted as an aftermath of a dysexecutive syndrome.

Roulet-Perez et al. [63] discussed left frontal lesions in children described by the combination of behavioral disorders, including aggressiveness, lack of inhibition, inattention and impulsiveness, and neuropsychological disorders, including difficulties in verbal and non-verbal reasoning, perseverations and reduction in verbal fluency. These symptoms are similar to those found in adult frontal syndrome, which includes impairment in executive functions and social behavior [63].

Debiais et al. [15] put forward a dysexecutive syndrome suggesting that the grammatical judgment assessed in their patients puts into play metalinguistic skills, which in turn are associated with executive functions. Moreover, problems in non-verbal tasks seen in the patients can also suggest an impairment in executive functions.

Hommet et al. [16] and Veggiotti et al. [20] reported similar behavioral patterns in their patients, including distraction and uninhibition, as well as neuropsychological ones, such as problems in abstraction, problem-solving, reasoning and perseveration. Veggioti et al. [20] proposed a dysexecutive syndrome in their patients, while their fundamental language, perception and spatial functions remained relatively unaffected. While clinical amnesia was not observed, their performance on memory tests was impaired, likely attributed to frontal regulatory disruptions, such as inattention and a lack of active learning and retrieval strategies. Similarly, Hommet et al. [16] put forward the dysexecutive syndrome to explain the persisting behavioral deficits and the problems seen in executing and maintaining strategies during language tests in adults.

##### Landau–Kleffner Syndrome

Majerus et al. [62] addressed the question of linking the short-term memory (STM) difficulties with phonological speech perception deficits by investigating posterior STG activation in three recovered patients with LKS. Specifically, these researchers aimed to investigate residual impairment in phonological STM. A single word repetition task (REP) and a task of repetition with four sequential words (MEM) were conducted during PET scans. In REP, both controls and LKS patients performed well. However, in MEM, patients DC and JPH showed significantly poorer performance than controls, particularly in the serial recall of the words, while patient TG’s performances were near normal. The two former patients with significant STM processing problems showed a reduced STM performance which was linked to decreased activation in the posterior STG, whereas TG’s better performance was associated with increased activity in this region. Based on this data, the authors support the notion that the STG appears to be activated exclusively for processing verbal information in STM, not for spatial or visual information. Finally, the authors considered whether the STM difficulties and reduced posterior STG activation in DC and JPH might be due to phonological speech perception deficits. However, since all patients showed normal single word perception, it’s unlikely that phonological identification issues explain their verbal STM difficulties.

In a second study by Majerus et al. [62], the dissociation between phonological and lexico-semantic STM and the implications for language processing were assessed among the same recovered LKS patients (TG, JPH and DC), who showed impaired performance in nonword immediate serial recall and a rhyme probe task, but performed normally on a category probe task. This study showed reduced phonological effects, such as word length and phonological similarity, indicating specific phonological processing impairments. TG displayed intact phonological and lexico-semantic processing but showed a clear dissociation between impaired phonological STM and preserved lexico-semantic STM. This dissociation was not solely due to residual phonological processing issues. The phonological and lexico-semantic STM task dissociations were not due to task difficulty, as controls found the category probe harder. JPH and DC had moderate phonological language processing deficits and mild lexico-semantic processing impairments, with a similar dissociation between impaired phonological STM and preserved lexico-semantic STM. JPH’s difficulties in speeded nonword repetition suggested a severe phonological STM deficit, as even simple two-syllable nonwords exceeded his STM capacity. Despite this, the phonological processes needed for the rhyme probe task were intact in both JPH and DC. The dissociations were based on both absolute performance and the impact of phonological and lexico-semantic information on STM, independent of task difficulty.

##### Dravet Syndrome

Thirty-seven patients had been longitudinally followed-up for a mean period of 6.3 years in a study by Ragona et al. [48]. Except for five patients with normal psychomotor development, all the rest exhibited mild to severe mental retardation and cognitive arrest associated with language impairment. At the last evaluation, performed at a mean age of 16 ± 6.9 years, mental retardation was present in 33 patients, associated with behavior disorders in 21, including attention deficit, hyperactivity, and opposition [48]. Individualized results about the adult patients were not presented in this paper. Similarly, Nabbout et al. [47] longitudinally studied 67 patients with DS, including both SCN1A mutated and non-mutated patients, (9 m–24 y) and found that DQ notably declined as age increased, transitioning from normal levels before the age of 2 to lower levels after reaching 3 years old. Hyperactivity and attention issues posed obstacles to learning, particularly up to the age of 6. Visuomotor skills were more significantly affected than language skills, which was attributed to impaired attention [47]. It should be noted that the results by Nabbout et al. [47] do not examine the adult participants separately. There is indirect evidence for a relation between language and cognition, as both language impairment and cognitive impairment have been reported to correlate with the same variables: persistence of seizures in adulthood, early onset of seizures and the presence of massive myoclonias and/or absences with myoclonias [42].

It has been suggested that the cognitive development of DS patients, the increasing cognitive gap between them and controls, and the increasing numbers of patients with mental retardation which increases along with age, does not truly reflect a regression or loss of skills, but rather a significant discrepancy between the chronological age of the patients and their mental age, which appears to remain stable [47,48,64,65]. A cognitive arrest has been identified to take place before the age of 5, and as the patients grow up, they are further burdened with behavior disorders and motor impairment, worsening their disability [48].

## 4. Discussion

This scoping review was set to investigate the research questions presented in the introduction section. In this section, we discuss the results in light of the research questions formulated.

**Q1.**  *Are there language deficits in adulthood across LKS, CSWS and Dravet syndrome?*

The findings collectively indicate significant variability in adults with encephalopathy. While there are studies in which impairments are not reported at any linguistic level in adulthood [35,54,55], other studies indicate severe linguistic deficits that significantly affect everyday communication [45,46]. However, collectively, the reviewed studies indicate the presence of linguistic deficits in adults with LKS, CSWS and Dravet syndrome to a significant degree.

**Q2.**  *If yes, in which modalities (oral production/oral comprehension/written production/written comprehension) and at which linguistic levels (pragmatics, phonology and morphosyntax, etc.) do they manifest?*

While oral production abilities were impaired across LKS, CSWS and Dravet syndrome, receptive abilities were not found to be affected for all [15,16,37,38,57]. Impairments concerned a range of expressive abilities affecting pragmatics and communication, lexical/semantic and syntactic abilities as well as oral motor abilities. In addition, deficient performance was shown at the word level, sentence level or even at the level of discourse and communication [15,16,19,20,33,34,35,37,45,46,52,54,55,56,57,58,59].

With respect to written language, there are a few studies that report difficulties and reveal symptoms of dyslexia and/or dysgraphia for these individuals [15,16,17,19,33,34,37,57].

**Q3.**  *Do these syndromes share the same manifestations of linguistic deficits or do they differ?*

These results indicate that different linguistic abilities are affected in these syndromes, which present variation in linguistic vulnerability. As the results indicated, language impairment the impacts of CSWS are predominantly on the domain of semantics and pragmatics, while grammar abilities are not completely spared in contrast to single word production, which is reported to stay intact. Interestingly, adults with CSWS have been reported to show written language difficulties at the production and reception level (dyslexia and dysgraphia) [16,19,33]. In addition, individuals with CSWS are reported to show difficulties in lexical or grammatical judgment tasks indicating deficient receptive abilities.

With respect to LKS, while recoverability of language abilities in adulthood has been reported, a significant number of adults with this syndrome show deficient performance in a wide range of abilities, including speech production, oral motor abilities [34,37,57], lexical/semantic abilities [57,58], phonology and impaired phonological discrimination [34,37,57] as well as expressive and receptive grammar [34,37,58]. Notably, LKS has been reported to result quite often in verbal auditory agnosia in childhood, a linguistic profile quite different than that of CSWS [15,17,20]. As far as Dravet syndrome is concerned, it is pointed out that language and communication abilities are severely affected. In the reviewed literature there were patients with significant impairment in basic communication skills [44,46,59,60] and various patients with no speech at all [45,59]. There is also significant impairment regarding oral motor skills, including dysarthria, deficits in motor programming and planning and parkinsonian traits observed in DS patients [59,60].

Apparently, the individuals with LKS, CSWS and Dravet syndrome differ not only in the domain which is affected, but also in severity in which the linguistic deficits are shown. Notably, just one comparative study was found that investigated in parallel deficits in CSWS and LKS [33]. These researchers point out that the clinical outcomes of these syndromes in adulthood are different and report distinct linguistic performances with CSWS individuals showing an absence of impairment in phonology or syntax and LKS individuals presenting striking difficulties with phonology and syntax. We underline that comparative studies can be very informative for the (dis)similarities among the syndromes. As no other between syndrome studies were found, direct comparisons are not possible. Therefore, the attempted comparisons in this section are subject to the limitations imposed by the reviewed literature.

**Q4.**  *Are there any cognitive correlates of the linguistic impairments in individuals with DEE/EE?*

While there are studies that indicate the co-existence of linguistic impairments with cognitive impairments, especially in the domain of executive functions, this is apparently not the case for all patients as dissociations between language and cognitive abilities were found in addition to associations.

Specifically, as far as the individuals with CSWS are concerned, it is pointed out that a dysexecutive syndrome has been described during the active phase of CSWS, interlacing metalinguistic abilities, executive functions and neuropsychological and behavioral deficits together, which significantly impacts linguistic performance [15,16,20,33]. LKS patients have been reported to present with dissociations between phonological short-term memory abilities and phonological and lexico-semantic processing [62]. As for DS patients, they have been consistently reported to present with mild to severe mental retardation and cognitive arrest related to language impairment. As they grow older, their disability becomes worse and behavioral problems and psychomotor deficits arise, and/or the differences between them and age-matched peers becomes more obvious [47,48]. In sum, it seems that the presence/absence of cognitive correlates for linguistic abilities and the specific form of interdependence (when language abilities are associated with cognitive ones) are syndrome dependent.

### Limitations

Our review process also suffers from some limitations that must be acknowledged. It is possible that focusing solely on PubMed and SCOPUS databases and English language studies may have excluded relevant research from papers in other languages. A limitation could also be that separate searches were conducted for each syndrome, although this was done in order to capture differences among the syndromes and to ensure that each syndrome was searched thoroughly.

However, in our view, this review contributes to the understanding of the linguistic deficits of speakers with developmental and epileptic encephalopathies, despite its limitations. First, it shows that encephalopathy significantly impacts language abilities, affecting the everyday life of these individuals in diverse ways. Second, it reveals that some of the studies reviewed do not even mention specific assessment materials as shown in Table S2 of the Supplementary Material [45,53,57,60]. Moreover, in all studies language assessment was conducted as part of a more general neuropsychological examination. It could be the case that tests that are linguistically informed reveal more detailed and granular profiles for each syndrome. Last but not least, as is shown in Table 1, many studies were single case studies or studies with very small sample sizes (*n* < 7) [16,19,20,34,38,56,57,58,60,62,66].

To sum up, despite the limitations of our own research, this review reveals the significance of studying the language abilities of large number of individuals suffering from encephalopathy, employing reliable psychometric materials.

## 5. Conclusions

This study aimed to shed light on an understudied topic; namely, the linguistic abilities of patients with developmental and epileptic encephalopathy. This scoping review confirms that the language outcome in adulthood has not been adequately studied, as the adult participants in these studies made up less than half of the combined number of participants. Examination of the literature revealed a large variability across patients and syndromes. For all syndromes there are patients with different levels of language abilities, although patients with DS are those with the worst outcomes, as a large number of them are non-verbal. Moreover, all modalities can be affected in all syndromes. This review reveals the need for more thorough scrutiny of the linguistic abilities, as most of these studies used diagnostic screening tests. Notably, these tests indicated language deficiencies to a great extent amongst the participants, which highlights that specific linguistic intervention techniques are needed and should be integrated within the behavioral treatment protocols of individuals with epileptic encephalopathy.

## Figures and Tables

**Table 1:** Characteristics of the studies included in the review.

Study	Paricipant Language	Only Adults	Design	# Participants	# Adults	Modality	Relation to Cognition	Minimum Age in Adults	Maximum Age in Adults	Age at Case Studies	SyndroMe	Follow Up or Not
Veggiotti et al. (2002) [19]	Italian	No	Case series	5	2	Both	No	19	23	-	CSWS	Follow up
Veggiotti et al., 2001 [20]	Italian	Yes	Case series	2	1	Both	Yes	-	-	19	CSWS	Follow up
Hommet et al., 2000 [16]	Not mentioned	Yes	Case Study	1	1	Both	Yes			19	CSWS	Follow up
Praline et al. (2003) [33]	French	Yes	Case series	7	7	Both	Yes	16*	26	-	CSWS & LKS	Follow up
Maltoni et al., 2016 [52]	Italian	No	Group	61	N.I.	Both	No	-	-		CSWS	Follow up
Seegmüller et al., 2012 [17]	French	No	Group	10	10	Both	Yes	20	29	-	CSWS	Follow up
Debiais et al., 2007 [15]	English	No	Group	10	2	Both	Yes	18,3	23,2	-	CSWS	No
Pera et al., 2013 [53]	Italian	No	Group	25	N.I.	Neuropsychological examination	Yes	-	31	-	CSWS	Follow up
Caraballo et al., 2014 [54]	Not mentioned	No	Group	29	13	Both	Yes	18.5	28	-	LKS	Follow up
Cockerell et al., 2011 [35]	Norwegian	No	Group	19	5	Both	Yes	22.3	29.1	-	LKS	Follow up
Duran et al., 2009 [55]	English	No	Group	7	4	Neuropsychological examination	No	20	27	-	LKS	Follow up
Pullens et al., 2015 [56]	Not mentioned	Yes	Case Study	1	1	Both	Yes	-	-	27	LKS	Follow up
Rejnö-Habte Selassie et al., 2010 [37]	Swedish	No	Group	19	8	Both	Yes	19.5	25.8	-	LKS	Follow up
Jokel & Meloff, 2020 [57]	English	Yes	Case Study	1	1	Both	No	-	-	48	LKS	Follow up
Sieratzki et al., 2001 [35]	English	Yes	Case Study	1	1	Both	Yes	-	-	27	LKS	Follow up
Stefanatos, 2008 [58]	Not mentioned	Yes	Case Study	1	1	Both	Yes	-	-	21	LKS	Follow up
Lévêque et al., 2020 [38]	Not mentioned	Yes	Case series	4	4	Comprehension	Yes	18	30	-	LKS	Follow up
Akiyama et al., 2010, [45]	Not mentioned	Yes	Group	31	31	Production	Yes	18	43	-	DS	Follow up
Genton et al., 2011 [46]	Not mentioned	Yes	Group	24	24	Production	Yes	20	50	-	DS	Follow up
Turner et al., 2017 [59]	English	No	Group	20	5	Production	Yes	23	28.5	-	DS	No
Darra et al., 2019 [42]	Italian	No	Group	84	50	Production	Yes	18	50	-	DS	Follow up
Kanatani et al., 2021 [60]	Not mentioned	Yes	Case series	2	2	Production	Yes	20	42	-	DS	Follow up
Brown et al., 2020 [61]	English	No	Group	45	7	Comprehension	Yes	N.I.	30	-	DS	No
Majerus et al., 2003 [62]	English	Yes	Case series	3	3	Both	Yes	18	20	-	LKS	No
Majerus et al., 2004 [62]	English	Yes	Case series	3	3	Both	Yes	28	20	-	LKS	No
Ragona et al., 2010 [48]	Italian	No	Group	37	N.I.	Neuropsychological examination	Yes	N.I.	28	-	DS	Follow up
Nabbout et al., 2003 [47]	Not mentioned, probably French	No	Group	67	N.I.	Production	Yes	N.I.	24	-	DS	Follow up

# means number of.

**Table 2:** Syndrome Characteristics.

Syndrome	Etiology	Cognitive Deficits	Language Deficits
Dravet	SCN1A gene mutation causing deterioration of function in at least 80% of cases—genetic factor Family history of epilepsy in 25–71% of cases	Developmental and cognitive delay apparent in the 2nd year of life Behavioral disorders, underdeveloped fine motor skills Mental impairment of various degrees	Delayed language acquisition Variability in oral production, ranging from minimally verbal to nonverbal and unintelligible speech production Variability in communication, ranging from mild to severe conversational speech intelligibility Speech disorders and impaired oral motor skills (including dysarthria, misarticulations, impaired prosody, impaired motor programming and planning and parkinsonian features) Comprehension and receptive abilities not extensively researched in adulthood
CSWS	Family history of epilepsy uncommon, in 10–15% of cases In a third of the cases, there are premorbid conditions, including structural defects	Only develops in the first 10 years of life Cognitive deterioration and behavioral disorders Cognitive and linguistic impairment either global or selective Motor impairment and decline in IQ and DQ in some patients	Consistent findings of deficits in pragmatics (production) Pragmatics and dysexecutive syndrome (cognitive function correlating with language) Deficits in semantic production and semantic fluency Naming preserved Syntactic production preserved in some patients, impaired in others Comprehension deficits reported in some patients Dyslexia, dysgraphia, poor writing (written language) Spelling and reading preserved in some patients, impaired in others (written language)
LKS	Unknown cause, genetic factors insignificant	Previously normal neuropsychological and cognitive development, age-appropriate language development, no lesions Language regression and progressive aphasia as primary symptoms Behavioral disorders Deficits in short-term auditory memory and verbal short-term memory impairment and language deficits of various grades in others	Auditory agnosia/unable to understand and distinguish sounds Variability in language outcomes in adulthood ranging from normal to poor Mild to severe language disorder, some patients unable to speak Moderate to severe communication problems due to impaired comprehension Oral motor deficits reported (stuttering, articulation and apraxia) Word finding problems, problems in phonological retrieval Impaired phonological discrimination (receptive abilities) Moderate to severe vocabulary and grammar deficits (receptive abilities) Variability in written language, slow reading and difficulties with phonetic reading in some patients

## Data Availability

Not applicable.

## Supplementary Materials

The following supporting information can be downloaded at: https://www.mdpi.com/article/10.3390/medicina60101635/s1, Figure S1: Number of studied languages; Figure S2: Number of group studies, case series and case studies; Figure S3: Number of studies addressing each syndrome; Figure S4: Number of studies addressing each modality; Figure S5: Number of studies addressing each modality for CSWS; Figure S6: Number of studies addressing each modality for LKS; Figure S7: Number of studies addressing each modality for DS; Table S1: Preferred Reporting Items for Systematic reviews and Meta-Analyses extension for Scoping Reviews (PRISMA-ScR) Checklist; Table S2: Summary of the Results. Reference [67] are cited in the supplementary materials.
