# Supplementary material for: How Encephalopathy Impacts Language Ability: A Scoping Review of the Linguistic Abilities of Adults with Developmental and Epileptic Encephalopathy
Source: Medicina (Kaunas). 2024 Oct 6;60(10):1635. doi: 10.3390/medicina60101635 (PMC11509453; doi:10.3390/medicina60101635)
Supplement: Supplementary file 1 [file medicina-60-01635-s001.zip › medicina-3207097-supplementary.pdf]

Supplementary Material

Table S1: Preferred Reporting Items for Systematic reviews and Meta-Analyses extension for Scoping Reviews (PRISMA-ScR) Checklist

SECTION	ITEM	PRISMA-ScR CHECKLIST ITEM	REPORTED ON PAGE #
**TITLE**			
Title	1	Identify the report as a scoping review.	p. 1
**ABSTRACT**			
Structured summary	2	Provide a structured summary that includes (as applicable): background, objectives, eligibility criteria, sources of evidence, charting methods, results, and conclusions that relate to the review questions and objectives.	p. 1
**INTRODUCTION**			
Rationale	3	Describe the rationale for the review in the context of what is already known. Explain why the review questions/objectives lend themselves to a scoping review approach.	p. 4-5
Objectives	4	Provide an explicit statement of the questions and objectives being addressed with reference to their key elements (e.g., population or participants, concepts, and context) or other relevant key elements used to conceptualize the review questions and/or objectives.	p. 5
**METHODS**			
Protocol and registration	5	Indicate whether a review protocol exists; state if and where it can be accessed (e.g., a Web address); and if available, provide registration information, including the registration number.	No registered protocol.
Eligibility criteria	6	Specify characteristics of the sources of evidence used as eligibility criteria (e.g., years considered, language, and publication status), and provide a rationale.	p. 5
Information sources*	7	Describe all information sources in the search (e.g., databases with dates of coverage and contact with authors to identify additional sources), as well as the date the most recent search was executed.	p. 5
Search	8	Present the full electronic search strategy for at least 1 database, including any limits used, such that it could be repeated.	p. 5
Selection of sources of evidence†	9	State the process for selecting sources of evidence (i.e., screening and eligibility) included in the scoping review.	p. 6
Data charting process‡	10	Describe the methods of charting data from the included sources of evidence (e.g., calibrated forms or forms that have been tested by the team before their use, and whether data charting was done independently or in duplicate) and any processes for obtaining and confirming data from investigators.	p. 6

SECTION	ITEM	PRISMA-ScR CHECKLIST ITEM	REPORTED ON PAGE #
Data items	11	List and define all variables for which data were sought and any assumptions and simplifications made.	p. 6
Critical appraisal of individual sources of evidence§	12	If done, provide a rationale for conducting a critical appraisal of included sources of evidence; describe the methods used and how this information was used in any data synthesis (if appropriate).	Not applicable
Synthesis of results	13	Describe the methods of handling and summarizing the data that were charted.	p. 6
**RESULTS**			
Selection of sources of evidence	14	Give numbers of sources of evidence screened, assessed for eligibility, and included in the review, with reasons for exclusions at each stage, ideally using a flow diagram.	p. 7 (flowchart)
Characteristics of sources of evidence	15	For each source of evidence, present characteristics for which data were charted and provide the citations.	Table 1
Critical appraisal within sources of evidence	16	If done, present data on critical appraisal of included sources of evidence (see item 12).	Not relevant for the review objectives
Results of individual sources of evidence	17	For each included source of evidence, present the relevant data that were charted that relate to the review questions and objectives.	Table 1
Synthesis of results	18	Summarize and/or present the charting results as they relate to the review questions and objectives.	Table S2 and sections *3.2.*
**DISCUSSION**			
Summary of evidence	19	Summarize the main results (including an overview of concepts, themes, and types of evidence available), link to the review questions and objectives, and consider the relevance to key groups.	Interim summary of section 3.2.1. and Section Discussion
Limitations	20	Discuss the limitations of the scoping review process.	Section 5. Limitation
Conclusions	21	Provide a general interpretation of the results with respect to the review questions and objectives, as well as potential implications and/or next steps.	Section Conclusions
**FUNDING**			
Funding	22	Describe sources of funding for the included sources of evidence, as well as sources of funding for the scoping review. Describe the role of the funders of the scoping review.	No funding

JBI = Joanna Briggs Institute; PRISMA-ScR = Preferred Reporting Items for Systematic reviews and Meta-Analyses extension for Scoping Reviews.

* Where *sources of evidence* (see second footnote) are compiled from, such as bibliographic databases, social media platforms, and Web sites.

† A more inclusive/heterogeneous term used to account for the different types of evidence or data sources (e.g., quantitative and/or qualitative research, expert opinion, and policy documents) that may be eligible in a scoping review as opposed to only studies. This is not to be confused with *information sources* (see first footnote).

‡ The frameworks by Arksey and O'Malley (6) and Levac and colleagues (7) and the JBI guidance (4, 5) refer to the process of data extraction in a scoping review as data charting.

§ The process of systematically examining research evidence to assess its validity, results, and relevance before using it to inform a decision. This term is used for items 12 and 19 instead of "risk of bias" (which is more applicable to systematic reviews of interventions) to include and acknowledge the various sources of evidence that may be used in a scoping review (e.g., quantitative and/or qualitative research, expert opinion, and policy document).

From: Tricco AC, Lillie E, Zarin W, O'Brien KK, Colquhoun H, Levac D, et al. PRISMA Extension for Scoping Reviews (PRISMAScR): Checklist and Explanation. Ann Intern Med. 2018;169:467–473. doi: 10.7326/M18-0850.

Table S2: Summary of the Results.

Authors, year	Method	Material	Language abilities assessed	Results
Veggiotti et al., 2002, 19	Neuropsychological assessment performed at latest follow-up, assessing language, memory and perception	Phonological profile in the Italian language, Boston naming test, token test, Peabody picture vocabulary test, sentence repetition test, multilingual aphasia examination, MT reading test, Battery for writing assessment at school for school achievement	Phonological processing and sentence structure analysis<br>Naming<br>Sentence comprehension<br>Word comprehension<br>Sentence repetition	• The severity and duration of CSWS varied among the patients.<br>• Outcomes categorized into three types: acquired frontal dementia, language deficits, and normal.<br>• Prognostic factors of the long-term outcome of CSWS were the length and age of onset of CSWS, the site of epileptiform activity, and the individual neuropsychological profile<br>• One adult patient normal language and one patient with preserved phonology and syntax but impaired semantics/pragmatics

Veggiotti et al., 2001, 20	Detailed neuropsychological assessment, observations and interviews	Phonological profile of the Italian language (PFLI)<br>Boston Naming Test<br>Token Test<br>Sentence Repetition test<br>Peabody Picture Vocabulary Test<br><br>Multilingual Aphasia Examination<br>MT reading test.<br>Battery for writing assessment in school children	Phonological processing and sentence structure analysis<br>Naming<br>Sentence comprehension<br>Word comprehension<br>Sentence repetition	Both patients:<br>• Minimal language production devoid of pragmatic and semantic content, phonologically and syntactically correct.<br>• Normal single-word comprehension and sentence repetition, but poor sentence comprehension.<br>• Poor naming with frequent semantic errors, suggesting a semantic rather than a visual-perceptual deficit.<br>• Reading age equivalent to a 7-year-old, with even poorer written word comprehension.<br>• Writing skills equivalent to a 6-year-old.
Hommet et al., 2000, 16	case study, longitudinal analysis of the clinical course, EEG recordings, and neuropsychological evaluations of behavior, language, and cognitive function	BDAE -Boston Diagnostic Aphasia	Discourse, word and sentence repetition, naming, syntax, oral comprehension, lexical fluency, reading, writing,	• Problems in writing, reading, sentence comprehension, lexical fluency, intact sentence production<br>• Fluctuation of the clinical course of the patient's condition, with temporary worsening or improvement of the paroxysmal abnormalities, epilepsy, and cognitive functions.<br>• Probably presence of a dysexecutive syndrome.

Praline et al., 2003, 33	oral and written language evaluation	Token Test, the Cardebat test, "l'Alouette"	Naming<br>Sentence comprehension<br>Categorical and phonemic fluency<br>Written language skills	• LKS patients: severe language disturbances and normal nonverbal IQ. Deficits in: word finding, reading, dictation and text reading. Error types: semantic paraphasias, visuospatial errors<br>• CSWS patients: normal scores in naming, deficit in verbal fluency for two patients, sentence comprehension, reading and writing.
Maltoni et al,, 2016, 52	neuropsychological tests	Test for mathematical and writing abilities (Giovanardi Rossi and Malaguti, 1994) verbal fluency test (Riva et al., 2000), the Token test<br>Raven's progressive matrices, Fepsy battery.<br>Recognition of words included in Fepsy battery (Alpherts, 1987)	Reading<br>Writing abilities<br>Verbal fluency<br>Syntactic comprehension<br>Abstract reasoning<br>Verbal working memory<br>Word recognition	• Longer Slow Wave Index (SWFI) duration correlated with lower scores in verbal IQ, performance IQ, abstract reasoning, visuoconstructional and visuospatial abilities, reading accuracy, and language semantic fluency.<br>• An earlier SWFI recording related to decreased language comprehension, visuoconstructional and visuospatial abilities after CSWS/ESWS disappearance

		Classification task included in Fepsy battery (Alpherts, 1987)		
Seegmüller et al., 2012, 17	neuropsychological tests exploring main cognitive functions	Rey Auditory Verbal Learning Test (RAVLT)	naming, verbal comprehension, reading	• The worst outcome in patients with prolonged global intellectual regression, better recovery in those with more specific and short-lived deficits<br>• Sequelae of Specific Language Impairment (SLI) in 1 patient. The rest performed well on naming tests, with residual verbal comprehension difficulties in 2.
Debiais et al., 2007, 15	Detailed language testing	Naming task, sentence completion task, oral comprehension task, reading speed task, Lexical Judgment and Grammatical Judgment tasks, Pictures task, experimental (not standardized) procedural discourse tasks and a narrative discourse task	Comprehension, lexical judgment, grammatical judgment, naming, expressive vocabulary and grammar, receptive vocabulary and grammar, reading, spelling, morphosyntax, pragmatics, metalinguistic abilities	• Language impairment equally severe in patients in remission and in an active phase of CSWS<br>• Deficits in pragmatic competence and impairment of executive functions.<br>• Pragmatics globally lower than the results seen in controls.<br>• 80–90% of the patients had impaired performance in morphosyntax and lexical performance.<br>• All subjects had normal performance in oral comprehension.

		(adapted from McDonald et al., 2000) (the Letter task, the Supermarket task)		
Pera et al., 2013, 53	Serial cognitive assessments	Specific tests are not mentioned	No specific language abilities assessed	• Cognitive decline in most patients during CSWS<br>• Outcomes varied by lesion presence and CSWS duration<br>• Worsening cognitive abilities overall<br>• Significant impact on language and performance tasks<br>• Permanent impairment in many children<br>• Improvement in patients without cerebral lesions after EEG regression<br><br>• Limited cognitive recovery in patients with lesions
Caraballo et al., 2014, 54	Various neuropsychological tests	The specific materials used are not included. Also, not all patients with LKS underwent full neuropsychological testing and thus, subtle deficits may not have been detected.	Comprehension, expression, repetition, naming	• Verbal auditory agnosia in all patients and severe aphasia in 24 patients and moderate in 5.<br>• Moderate nonlinguistic cognitive dysfunctions in 14 patients.<br>• Behavioral disturbances found in 16 patients.<br>• 8 patients recovered language completely. The rest still face language deficits of different degrees.

Cockerell et al., 2011, 35	Language assessment, neuropsychological assessment, evaluation of behavioral and social interactions, semi structured interviews with the parents	Language tests from KOAP (surveying processes regarding decoding of words), and SIT (impressive language test of children). After 2005 and at follow-up; Clinical Evaluation of Language 4 Fundamentals (CELF-4), the Test for Reception of Grammar (TROG), and the Peabody Picture Vocabulary Scale III.	Language form, content, use, grammar, receptive language skills, vocabulary	• Auditory agnosia in 7 patients, verbal agnosia in 2 and auditory discrimination deficit in 10<br>• After 10.8 years, 21% had normal language, 58% had functional language with some auditory and verbal issues, and 21% relied on sign language.<br>• All patients improved in language or used sign language for communication.<br>• Later onset, short initial aphasic episodes, and speech ability fluctuations correlated with better future language skills.

Duran et al., 2009, 55	clinical and psychological examination	Overall, language skills were evaluated through interviews, assessment scales, and observation of language disturbances and limitations in patients with LKS.	The study does not mention the specific language abilities that were examined.	• LKS patients often have lower quality of life due to language challenges affecting socialization, daily skills, and adaptation. . • Continued epileptiform activity on EEG and resistant seizures may suggest poorer language outcomes for LKS patients. • Complete resolution of language disturbances in 1 patient, gradual improvement in 3 • Three patients still exhibited aphasia and verbal auditory agnosia. • Limited progress in language development in the patient with delayed onset of language dysfunction • Only 1 patient reported a satisfactory quality of life
Pullens et al., 2015, 56	Case study, language assessment	The specific language tests are not mentioned	Language comprehension and production, expressive language, abstraction of the inner language, articulation, written language	• Patient shows uneven auditory response in left and right cortex, indicating altered language pathways. However, intact integration of letters and sounds in superior temporal gyrus. • Continues to struggle with sentence structure, word formation, intonation, and learning new vocabulary orally.
Rejnö-Habte Selassie et al., 2010, 37	neuropsychological assessment, speech and language assessment, auditory assessment	Language: Peabody Picture Vocabulary Test, NEPSY, TROG-2, the Clinical Evaluation of	Oral motor abilities, articulation, receptive vocabulary, word retrieval, receptive and expressive grammar,	•Speech and language dysfunction at follow-up in more than half of the participants • IQ levels, working memory, and processing speed also affected. • Dysfunction of communication, oral motor ability, and stuttering noted in a few.

		Language Fundamentals, the FAS test<br><br>Communication: Asperger Syndrome Screening Questionnaire (ASSQ)	phonology and spelling	
Jokel and Meloff, 2020, 57	Case study, linguistic assessment, interviews	Specific tests are not mentioned.	Spontaneous speech, receptive vocabulary / auditory recognition of words, naming, grammar and syntax, oral reading, comprehension of written language, abstract thinking, semantics	• Comprehension problems<br>• Patient reliant on lip-reading<br>• Slow reading with misarticulations and omissions<br>• Difficulty in naming, 'deaf speech' characteristics<br>• Limited vocabulary, intact auditory recognition<br>• Struggling with the comprehension of grammatically complex sentences
Sieratzki et al., 2001, 35	Case study, language assessment	PALPA and the Test of Reception of Grammar (TROG), lip reading tests and tests of	Language comprehension, lip reading, productive vocabulary and articulation in English and BSL,	• Severe restrictions in comprehension and production of spoken English as well as lip-reading, while reading was comparatively less impaired. BSL was by far the most efficient mode of communication.

		recognition of consonant-vowel syllables, Snodgrass and Vanderwart picture set For BSL: BSL vocabulary comprehension test, a non-standardized narrative production task	comprehension of BSL vocabulary and grammar, production of BSL narrative, reading	
Stefanatos, 2008, 58	Case study, Neuropsycholoigical evaluation, language assessment	Confrontation naming Paragraph reading, multiple-choice questions	Sign language, verbal speech, pitch, volume, vocal rate, naming, repetition, verbal production, comprehension of auditory language, reading comprehension	• Poorly articulated single words, simple objects in confrontation naming, poor repetition, good reading skills, normal visual memory, significant disturbance in environmental sound recognition<br>• Case study suggests temporal lobe issues with sound processing of rapid frequency changes, impacting speech analysis of crucial frequency cues.
Lévêque et al., 2020, 38	Listening, audiometric, prosodic and musical tests	Pitch Discrimination Task. Two Auditory short-term memory tests adapted from Tillmann et	Basic perceptual skills, production skills, pitch discrimination, prosody, speech prosody recognition	• 3/ 4 patients exhibited amusia, with difficulties in pitch discrimination and memory for melody and rhythm<br>• Epilepsy affects sound processing networks beyond speech issues, even after electrophysiological normalization..

		al. (2009), one with tones, the other with spoken syllables Two subtests of the receptive prosody test from the Montreal Evaluation of Communication (MEC) protocol		
Akiyama et al., 2010, 45	observations	Specific tests are not mentioned.	Oral communication, reading	• 7 patients were nonverbal.<br>• 9 patients could speak only a few words.<br>• 9 patients could hold basic conversations.<br>• 5 patients could engage in simple conversations and read to some extent, with IQs ranging from below 40 to 50 for four of them.<br>• 1 patient with borderline DS had milder intellectual disability, lived independently, but developed psychosis.
Genton et al., 2011, 46	cognitive/behavioral and social examination	Specific tests are not mentioned. Specific quantitative assessment beyond childhood were not performed.	Verbal communication	• Severe to moderate mental retardation, significant language impairment in 21 patients.<br>• No language in 3, non-communicative language in 4, poorly structured but communicative language in 7, and poor but communicative language in 7.

Turner et al., 2017, 59	Speech and language assessment	Complexity of Communication Scale, Early Motor Control Scales or Verbal Motor Production Assessment for Children, Frenchay Dysarthria Assessment, Diagnostic Evaluation of Articulation and Phonology (DEAP), Preschool Language Scales or Clinical Evaluation of Language Fundamentals, Peabody Picture Vocabulary Test, Expressive	Receptive and expressive language, conversation/oral communication, vocabulary, grammar, articulation, prosody, intelligibility	• Speech issues in group V: severe intelligibility issues in 3, moderate in 4, mild in 5, and normal in 1. Issues include imprecise articulation, abnormal nasal sound, breathy voice, low pitch, and prosodic errors. • Language testing in 13 patients: severe impairment in both receptive and expressive language noted in 9 out of 13.

		Vocabulary Test, Test For Reception of Grammar(TROG). A tailored comprehensive assessment battery for V and MV patients. A tailored perceptual speech assessment in verbal patients with conversational speech for V patients.		
Darra et al., 2019, 42	cognitive evaluation, language assessment, behavioral assessment, clinical observation	No tests specifically for language	Expressive language/ oral communication	•Correlation of the complete phenotype with the severity of cognitive, language, and neurological impairment<br>• Language skills varied (Normal in 8 cases, simple conversations sustained by 7 patients, short sentences used by 12 patients, only isolated words produced by 11 patients, 12 patients not capable of speaking)

Kanatani et al., 2021, 60	Case study, observation	No specific materials used.	Speech, communication and intelligibility, based on observation and neurological examination	• Unclear whether the parkinsonism in DS is a consequence of severe mutations in the SCN1A gene or the result of intractable seizures and the use of many kinds of AEDs at high doses for long periods.<br>• The motor symptoms did not respond to levodopa, making it challenging to attribute these symptoms directly to parkinsonism
Brown et al., 2020, 61	Standardized neuropsychological testing	The NEPSY-II — Affect Recognition, The NEPSY-II — Comprehension of Instructions, The Delis–Kaplan Executive Function System (D-KEFS) — Verbal Fluency, The Social Communication Questionnaire (SCQ)	Receptive language, comprehension of instructions, letter fluency (phonetic fluency), category fluency (semantic fluency), and category switching	• All performances on tests on working memory, executive functions and verbal skills impaired<br>• No strong evidence for preservation of verbal skills (e.g. 3 scored in average range, 3 below average on Vocabulary).
Majerus et al., 2003, 63	phonological and lexico-semantic language and STM during PET scan	Two different verbal repetition tasks: single word repetition (REP) and repetition of	Phonological language processing	• In REP task: Both controls and LKS patients performed well<br>• In MEM task: Significantly poorer performance in 2 patients, compared with controls. The other patient's performance was nearly normal.

		sequences of four words (MEM).240 French words from the Brulex database (Content et al., 1990)		• Reduced STM performance in the 2 patients was linked to decreased activation in posterior STG.<br>• Better performance linked to increased activity in posterior STG.<br>• It is possible that STG activates exclusively for verbal information in STM, not spatial or visual.<br>• STM difficulties in the 2 patients unlikely due to phonological speech perception deficits.<br>• Normal single word perception across all patients.
Majerus et al., 2004, 64	short term memory measures, language measures	Immediate Serial Recall (ISR), Digit Span, Category Probe Task (lexico-semantic STM), Word Length Effect Task, Phonological Similarity Effect Task, Phonotactic Frequency Effect, Lexicality Effect, Word Imageability Effect, Rhyme and Semantic Category Probe	Phonological language processing, lexico-semantic language processing	• Clear dissociations between phonological and lexico-semantic STM measures in all 3 patients. Impaired performance in nonword immediate serial recall and in a rhyme probe task, normal performance in a semantic category probe task<br>• Reduced phonological effects, e.g. word length and phonological similarity, indicating specific impairments in phonological processing<br>• No consistent relationships between impairments in phonological and lexico-semantic STM and their respective processing abilities found.

		Tasks, French Peabody Picture Vocabulary Test E.CO.S.SE (French adaptation of TROG), Speeded Non-Word Repetition Task (phonological processing), Phonological Awareness Task (adapted from ISADYLE), Speeded Word Repetition (lexico-semantic processing), Categorization of Low and High Imageability Words		
Ragona et al., 2010, 48	Cognitive assessment, behavioral assessment	No tests used specifically for language	No specific language abilities assessed	• Cognitive impairment and behavior disorders common in DS patients<br>• Mental retardation present in the majority of cases, often associated with attention deficit, hyperactivity, and oppositional behavior

				• Largely poor cognitive outcome in DS patients<br>• Increase in severe mental retardation with age, reflects cognitive development arrest before age five
Nabbout et al., 2013, 47	neuropsychological evaluation, parents' interviews, behavioral observations	No tests specifically for language.<br>A semi-quantitative psychomotor scale (SQPS) (no/moderate/severe delay) for global, motor and language development	Verbal communication	• Language acquired by all patients. Patients over six years old could construct sentences.<br>• SCN1A mutation contributes to cognitive delay, separate from epilepsy.<br>• Better gait and language skills in non-mutated patients.<br>• DQ/IQ drops from normal before age 2 to low after age 3.<br>• Hyperactivity and attention disorders hinder learning up to age 6.<br>• Speech development faster than visuomotor function.

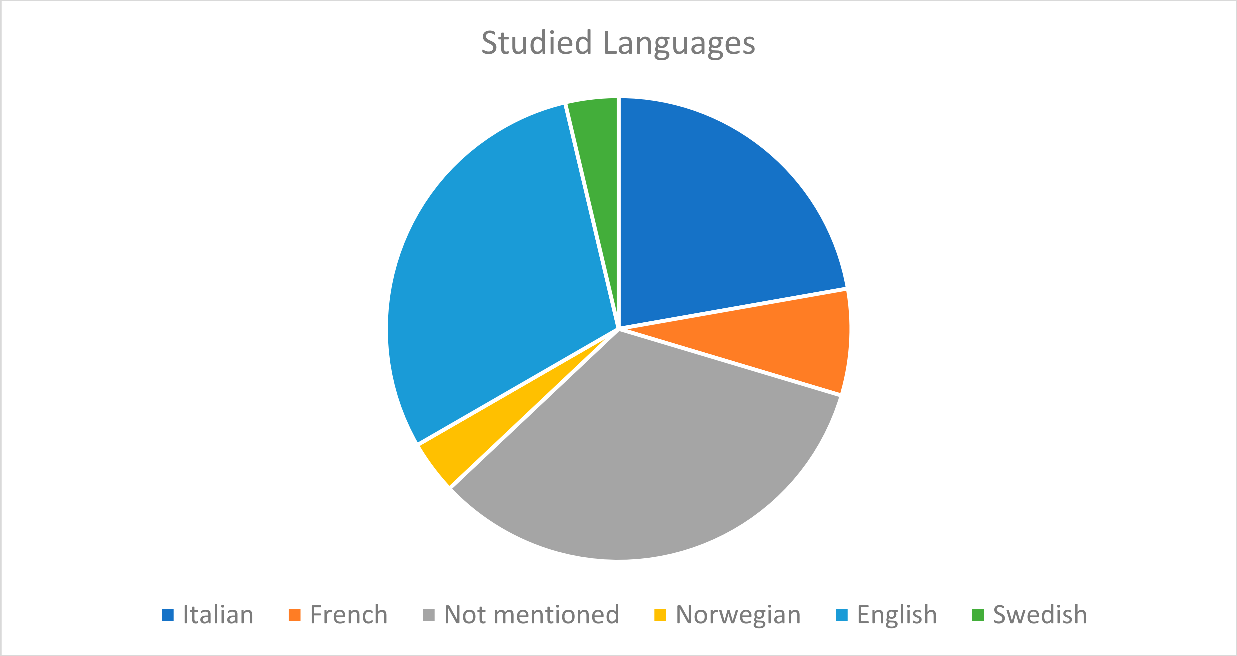

Figure S1. Number of studied languages

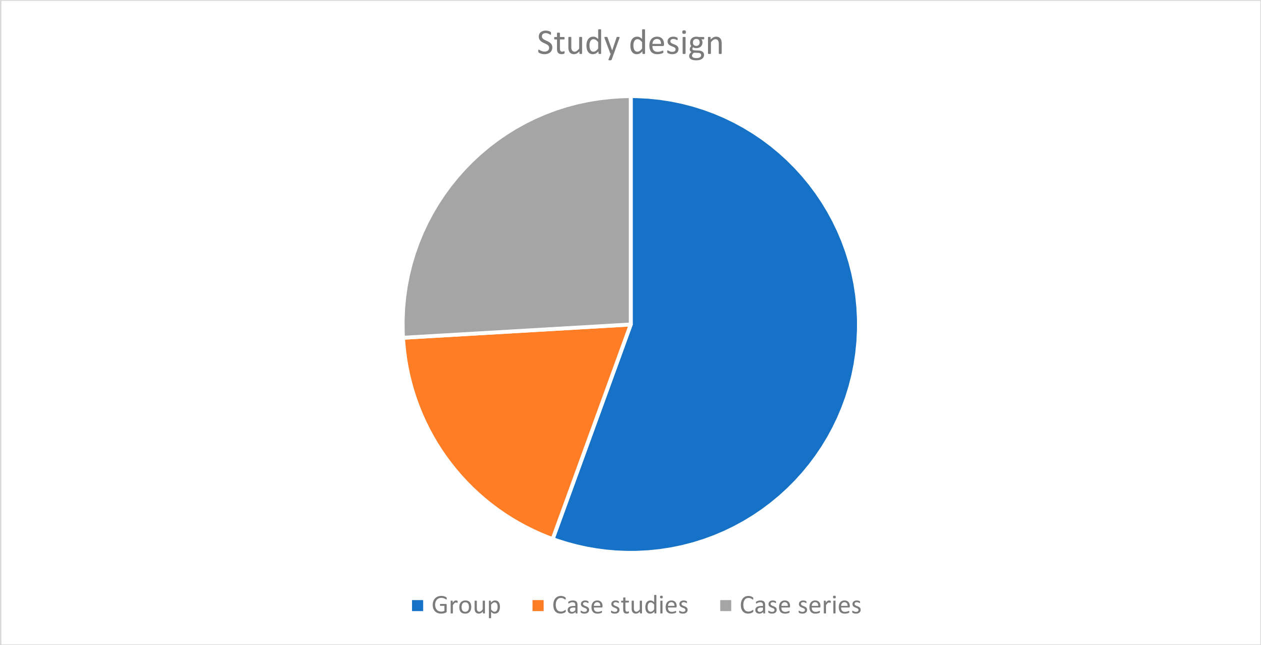

Figure S2: Number of group studies, case series and case studies

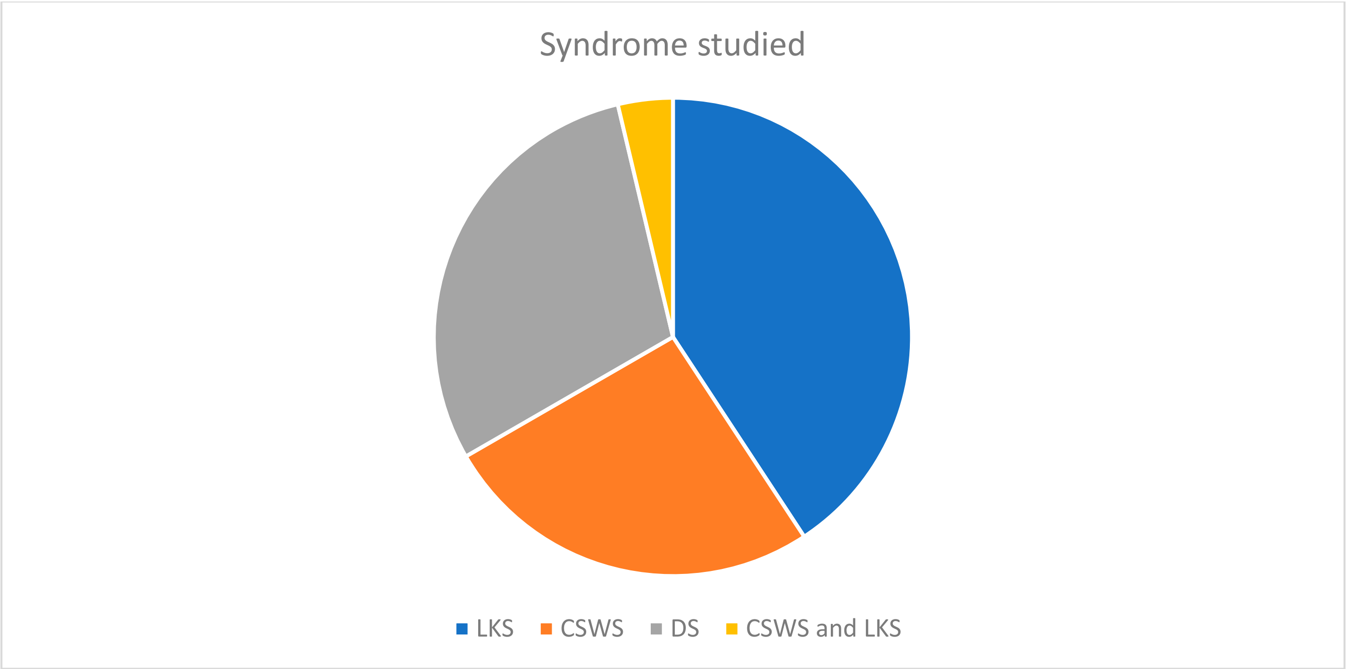

Figure S3: Number of studies addressing each syndrome

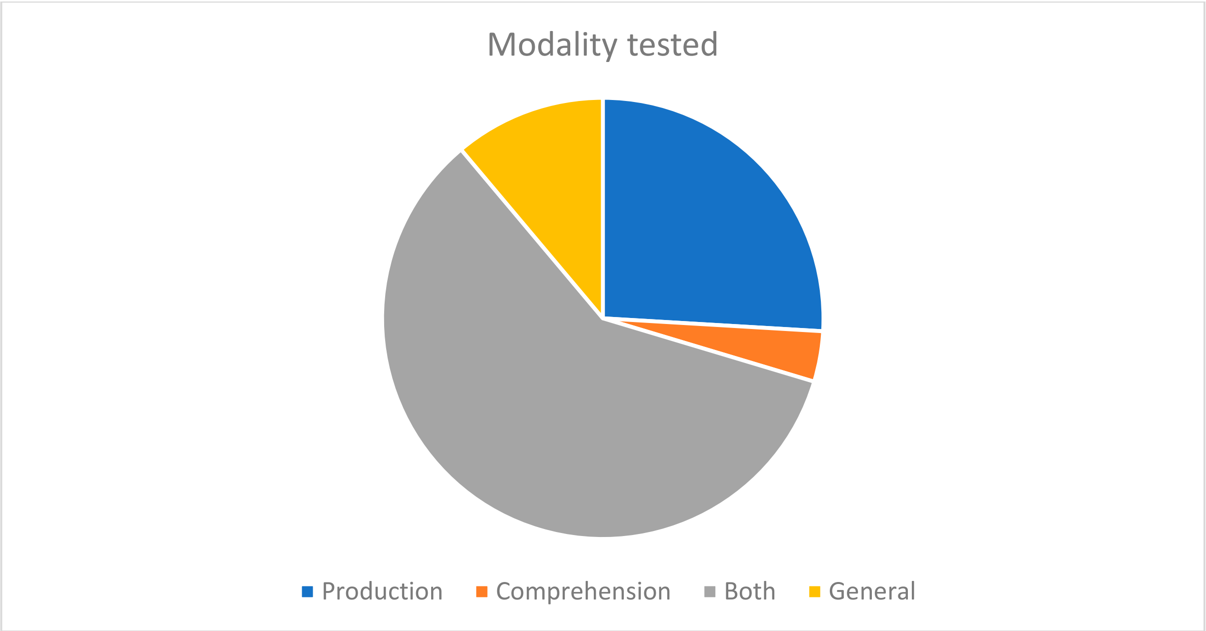

Figure S4: Number of studies addressing each modality. General: no information about specific tests and modality tested.

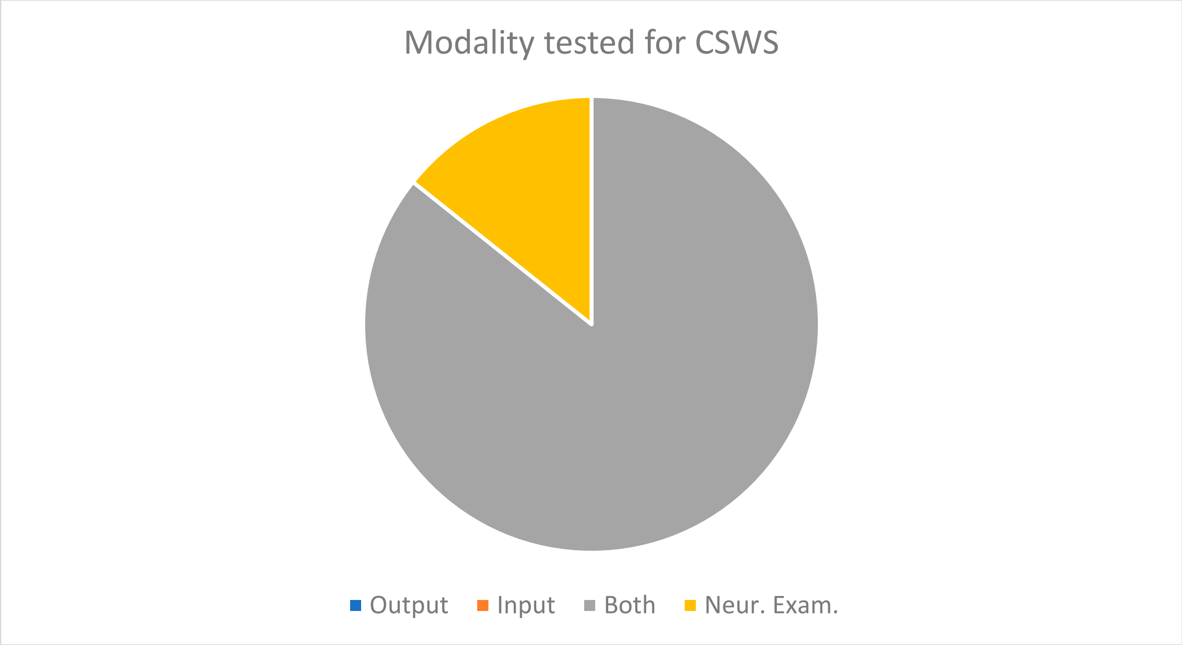

Figure S5: Number of studies addressing each modality for CSWS

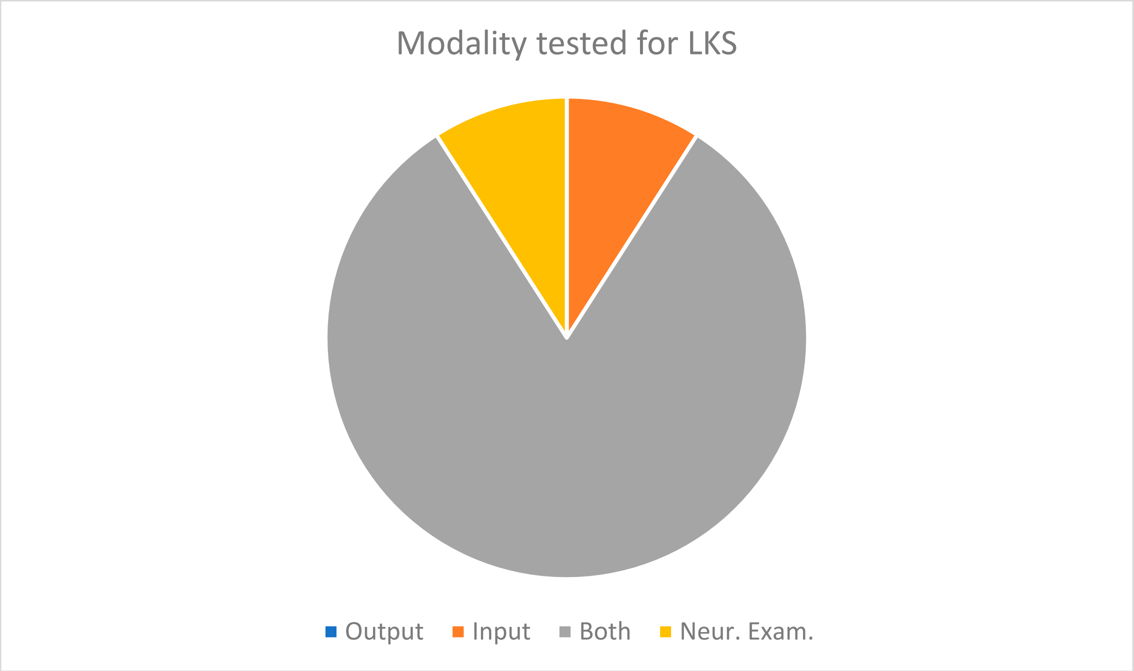

Figure S6: Number of studies addressing each modality for LKS

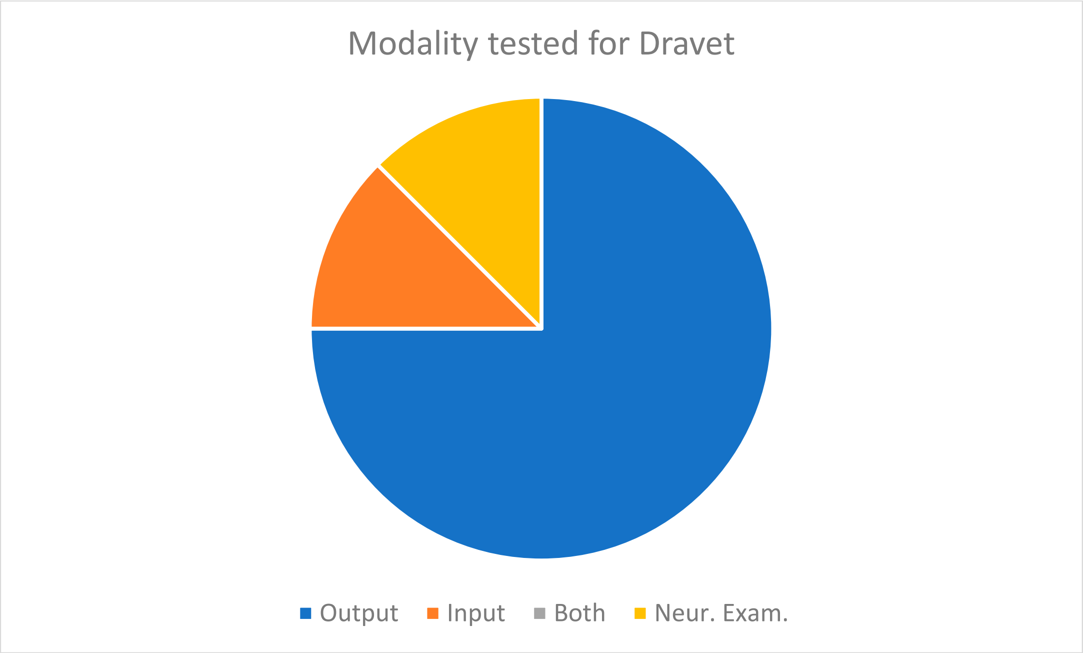

Figure S7: Number of studies addressing each modality for DS
